# Kidney Diseases

**Published:** 1895-05-11

**Authors:** 


### PROGRESS IN MEDICINE.

### KIDNEY DISEASES.

(*Continued from page* 45).

Pavy[18] replies that sugar is normally present to the amount of ·05 grains per cent. because fermentation takes place to some extent on the addition of yeast, and that crystals are often formed by phenyl hydrazine. A. H. Allen confirms[19] this view, and has constantly found sugar after kreatinine has been removed by Johnson's new method from normal urine. It seems unfortunate that these high authorities differ as to facts observed. A comparison of the different tests for the presence of bile colouring matter in the urine has been made by A. Jolles on account of their importance as the first indication of obstruction in the bile ducts. Taking solutions of bile in urine of known strength he found that Gmellin's and fifteen other tests were by no means delicate, and he recommends that a drop of hydrochloric acid with an excess of chloride of barium and 5 c.c. of chloroform be added to 50 c.c. of urine.[20] He allows the mixture to settle, pours off the liquid, dries the residue, and adds three drops of sulphuretted sulphuric acid or nitric acid, when the characteristic coloured rings will appear.

Rosin advises that a 3 c.c. of a 10 per cent. mixture of the tincture of iodine in alcohol be poured on the top of the urine when a green ring will show the presence of bile pigment.[21] The presence of antipyrin, however, gives the same result with iodine.

"Transitory Albuminuria" is the subject of papers by Krauss and Hwass.[22] The latter found it in 15 per cent. of the 559 soldiers whom he tested, while hyaline and granular casts were found even in some cases where no albumen was present. Krauss denies that it has any tendency to develop into Bright's disease or to shorten life. Boyd has discussed with great care the question whether albuminuria is caused by filtration through an injured membrane or by a depraved activity of the glomerular epithelium. He denies that albumen normally passes out of the glomeruli and is absorbed by the tubular epithelium, saying that none is found in healthy kidneys if plunged in boiling water. Moreover altered pressure of blood will not account for the presence of albumen in the urine. The amount of proteids present is often higher than in mere transudations, nor is there any correspondence between the albumen quotient in the blood and in the urine, and the variations of the two proteids found in the urine are too great to be accounted for by mere alterations of the blood pressure.[23] Hence he decides against the mechanical filtration theory. A review of the whole subject is given by an American writer, who shows that the old arguments from Nussbaum's experiments really tell against the filtration theory as long as the cells are intact.[24] He concludes that albumen is excreted chiefly by the tubular epithelium, and to a less extent by filtration from the glomeruli, when these have become the seat of nephritis.

The toxicity of the urine has been investigated by Lapicque, Marette, and Roger.[25] The former discovers that a pure milk diet increases this toxicity, while one of rice and milk lowers it. Excessive intestinal fermentation or fatigue increase it, but ordinary muscular effort or the changes in normal diet have no effect. The poison is destroyed by boiling, and has no relation to the colour of the urine. Roger shows that it is not dialysable, while urea and other products which pass through a membrane are comparatively inert.[26] These toxins lower the body temperature, and produce coma, diarrhœa, hæmorrhage, and death in a few hours. Strassers finds that phenol is normally formed and excreted to the amount of five to seven centigrammes daily, but that in early typhoid, during the resolution of pneumonia, suppuration and leucocythæmia a larger amount is poured out.[27] An increase does not imply necessarily an increase of intestinal putrefaction.

The nucleins or glycerised phosphates produced by the polynuclear white corpuscles are represented by Aulde as the antiseptics by which the body protects every spot which is exposed to bacterial attacks.[28] He finds that in Bright's diseases, as well as in anæmia, dyspepsia, malaria, and pulmonary diseases, the artificial addition of nucleins to the blood enables the tissues by stimulation of the leucocytes to combat the disease. This "defensive proteid" appears to be non-toxic, and can be given in doses of one-third minim and upwards every two hours in acute diseases. Vaughan prepares it from brewer's yeast, but it is obtained also from blood glands, the marrow, &c.

The indications given by the presence of indican in the urine are much debated. Beckman concludes from the examination of forty cases that increased indican formation cannot be depended on as evidence of concealed suppuration, and that there is no proof of its formation elsewhere than in the intestine. Indeed, the pyogenic germs found in ordinary abscesses do not belong to the class which produce indican in albuminous media.

A review of the question, as far as relates to infantile tuberculosis, is given in "L'Union Medicale," October 23rd. Steffin found it in a very large proportion of tubercular cases, but less frequently than in measles. Djouritch finds it constantly in infantile tuberculosis, and regards it as a valuable test.[30] She agitates equal parts of urine and hydrochloric acid with chloroform. The latter takes up the colouring matter which then forms a violet layer.

The death of the Czar recalls the paper read by Clifford Allbut on mental anxiety as a cause of granular kidney, in which he showed what a large proportion of his cases had gone through prolonged mental strain.[31] Similar remarks are quoted from Dickinson and Sir G. Johnson. The post-mortem on the Czar showed chronic interstitial nephritis with atrophy of the cortex. There was an infarct in the left lung, and the heart was hypertrophied, and its muscle degenerated. Zacharin writes to the *Lancet* that he had had three attacks of influenza, but albumen appeared in July with casts and enlargement of the heart.[32] That the disease existed only for a short time is shown by the easily separable capsules of the kidneys, and their slight density. Fatigue and exposure to cold and damp appear to have been among the chief causes of his illness.

[18] Lancet, July 18th. [19] Lancet, July 28th. [20] Centralb. f. Med. Weiss., May 19th. [21] Amer. Med. and Surg. Bulletin, Oct. 1st. [22] Edin. Med. Journ., Oct. [23] Edin. Med. Journ., Sept. [24] Amer. Med. and Surg. Bulletin, Oct. [25] Med. Weekly, July 27th. [26] Lancet, June 30th. [27] Med. Record, March 16th. [28] New York Med. Journ., Sept. 29th. [29] Amer. Med. and Surg. Bulletin [30] Lancet, Nov. 24th. [31] Brit. Med. Journ., Nov. 3rd. [32] Lancet, Dec. 1st.

